# Identification of prognostic gene expression signatures based on the tumor microenvironment characterization of gastric cancer

**DOI:** 10.3389/fimmu.2022.983632

**Published:** 2022-08-12

**Authors:** Qingqing Sang, Wentao Dai, Junxian Yu, Yunqin Chen, Zhiyuan Fan, Jixiang Liu, Fangyuan Li, Jianfang Li, Xiongyan Wu, Junyi Hou, Beiqin Yu, Haoran Feng, Zheng-Gang Zhu, Liping Su, Yuan-Yuan Li, Bingya Liu

**Affiliations:** ^1^ Department of General Surgery, Ruijin Hospital, Shanghai Jiao Tong University School of Medicine, Shanghai, China; ^2^ NHC Key Lab of Reproduction Regulation (Shanghai Institute for Biomedical and Pharmaceutical Technologies), Fudan University, Shanghai, China; ^3^ Shanghai Engineering Research Center of Pharmaceutical Translation, Shanghai, China

**Keywords:** gastric cancer, tumor microenvironment, prognostic model, CTGF, fibroblasts

## Abstract

Increasing evidence has elucidated that the tumor microenvironment (TME) shows a strong association with tumor progression and therapeutic outcome. We comprehensively estimated the TME infiltration patterns of 111 gastric cancer (GC) and 21 normal stomach mucosa samples based on bulk transcriptomic profiles based on which GC could be clustered as three subtypes, TME-Stromal, TME-Mix, and TME-Immune. The expression data of TME-relevant genes were utilized to build a GC prognostic model—GC_Score. Among the three GC TME subtypes, TME-Stomal displayed the worst prognosis and the highest GC_Score, while TME-Immune had the best prognosis and the lowest GC_Score. Connective tissue growth factor (CTGF), the highest weighted gene in the GC_Score, was found to be overexpressed in GC. In addition, CTGF exhibited a significant correlation with the abundance of fibroblasts. CTGF has the potential to induce transdifferentiation of peritumoral fibroblasts (PTFs) to cancer-associated fibroblasts (CAFs). Beyond characterizing TME subtypes associated with clinical outcomes, we correlated TME infiltration to molecular features and explored their functional relevance, which helps to get a better understanding of carcinogenesis and therapeutic response and provide novel strategies for tumor treatments.

## Introduction

Gastric cancer (GC) is responsible for over 1 million new cases in 2020 and an estimated 0.77 million deaths, incidence ranked fifth, and mortality ranked fourth, as proposed by WHO in 2020 (1). In addition to tumor cells, cancer tissue is also composed of numerous distinct non-cancerous cell types. Together, these are termed as the tumor microenvironment (TME). Increasing evidence has elucidated a crucial role of the TME in carcinogenesis and therapeutic response (2). Exploring biomarkers in the scenario of the TME will provide new ideas for predicting prognosis and developing novel therapeutic strategies.

In the past several decades, TNM staging system has been playing an important role in the clinical practice of GC; however, it is difficult to explain the patients with the same TNM stages and similar treatment options but different clinical outcomes. Some studies have explored the significance of dysregulated signaling events in both GC cells and environment cells (3), suggesting that the TME infiltration pattern has predictive power for clinical outcomes and GC subtyping based on the TME could be a complement to current staging methods.

A series of computational tools, such as CIBERSORT (4), MCP-counter (5), TIMER (6), xCell (7), EPIC (8), and quanTIseq (9), designed for estimating the abundance of various cell populations based on bulk transcriptome, provide more accessible opportunities to decipher the TME infiltration. In a recent study (10), the CIBERSORT algorithm and Microenvironment Cell Populations-counter method were applied to bulk transcriptomic data of GC patients by which the abundance of 22 types of immune cells and two types of stromal cells was estimated, and three TME phenotypes were eventually defined based on the landscape of the GC TME infiltration. However, quite a number of cell types were ignored due to the restriction of algorithms. By integrating the advantages of gene set enrichment with deconvolution approaches, xCell (7) accommodates the most comprehensive cell types, a total of 64 immune and stromal cell types, including a variety of adaptive and innate immune cells (lymphoids and myeloid), hematopoietic progenitor cells, epithelial cells, and extracellular matrix (ECM) cells.

In our previous work, the gene expression profiles of 111 GC and 21 normal samples in the Chinese population were examined (GSE54129); meanwhile, the clinical information was collected, and follow-up was carried out for 13 years. In this study, we comprehensively calculated the TME infiltration patterns of 111 tumor and 21 normal samples based on bulk transcriptomic profiles by using xCell. Based on the xCell scores, three GC TME subtypes with distinct survival outcomes were then obtained. We further built a GC prognostic model, GC_Score, with the expression level of TME-relevant genes. The higher the patient’s GC_Score, the worse the survival and the lower the drug sensitivity as well. Fibroblasts, which were significantly correlated to the GC_Score, and connective tissue growth factor (CTGF), the highest weighted gene in the GC_Score model, were taken to the subsequent study. CTGF, also known as cellular communication network 2 (CCN2), is a member of the CCN (CCN1-6) family proteins (11). CTGF plays a role in diverse biological processes including tumorigenesis and fibrosis (12), regulates diverse cellular processes including ECM protein synthesis, adhesion, proliferation, and apoptosis through its diverse interacting partners, and thus affects developmental and pathological processes ranging from fibrosis, progenitor cell fate decisions, angiogenesis, to inflammation and tumorigenesis (13, 14). The increased CTGF expression was observed in GC, and a high CTGF expression was associated with poor clinical outcomes. By examining the marker gene expression at transcript and protein levels, CTGF was proven to have the potential to induce transdifferentiation of peritumoral fibroblasts (PTFs) to cancer-associated fibroblasts (CAFs), which had been shown to actively communicate with cancer cells and contribute to tumor progression.

## Materials and methods

### Data source

The gene expression profile dataset GSE54129 (tissues from 111 GC and 21 normal samples, Ruijin cohort) was from the platform of Affymetrix HG-U133_Plus_2. All patients provided a written informed consent (IC). Ethics committees approved the collection of samples. The clinicopathologic information including Gender, Age, pTNM stage, Histological type, Borrmann classification, Tumor location, Differentiation, Tumor invasion, Regional lymph node, and Distant metastasis and 13 years of follow-up were collected.

### Characterization of cell-type proportions in the tumor microenvironment

xCell R package was used to calculate the proportion of cell populations based on bulk gene expression data (7). xCell integrates single-sample gene set enrichment analysis (ssGSEA) and deconvolution approaches and allows the enumeration of 64 cell types, which were divided into five cell type clusters: Stromal, Epithelial, Lymphoid, Myeloid, and hematopoietic stem cells (HSCs).

### Construction of the GC_score model

The limma package (15) was used to identify differentially expressed genes (DEGs) with the cutoff of |log fold change| >1.5 and Benjamini–Hochberg-adjusted p < 0.01. In order to investigate the prognostic significance of individual DEGs, Kaplan–Meier survival analysis was performed, and genes with log-rank p < 0.05 in both overall survival (OS) and disease-free survival (DFS) were taken as independent prognostic biomarkers to build the GC_Score model.

The samples with survival information available were randomly divided into a training set and a test set by the ratio of 7:3. Glmnet R package was adopted to build the GC_Score model. The most significant prognostic markers were selected using the penalized Cox regression model with least absolute shrinkage and selection operator (LASSO) penalty, and the optimal values of penalty parameter λ were determined by 10-fold cross-validations in the training set (16).

Receiver operating characteristic curve (ROC) curves were used to assess the sensitivity and specificity for survival prediction based on the GC_Score (17).

### Drug sensitivity analysis

The pRRophetic R package (18) was used to predict clinical drug response according to tumor gene expression data, which was achieved with statistical models based on gene expression and drug sensitivity data in a large panel of cancer cell lines.

### Isolation and purification of cancer-associated fibroblasts and peritumoral fibroblasts

Human tumor tissues and their non-tumor tissues were obtained from GC patients who underwent surgical resection at the Ruijin Hospital (Shanghai, China). The tumor and peritumoral tissues of GC were minced into small pieces and digested in dulbecco's modified eagle medium (DMEM) supplemented with 10% fetal bovine serum, 400 U/ml collagenase IV, and 1% penicillin/streptomycin at 37°C for 1 h. After centrifuging for 15 min at 1,100 rpm, the suspension was washed twice with DMEM. Following manufacturer’s instructions, anti-fibroblast MicroBeads (MiltenyiBiotec) were used to isolate fibroblasts from the cell pellet.

### Cell culture

DMEM supplemented with 20% fetal bovine serum (Gibco), 1% penicillin/streptomycin (Yeasen), and 1% penicillin/streptomycin (Yeasen) was used for resuspending the fibroblasts. All experiments were conducted with cells from passages 3–6. Fibroblasts were plated in 35-mm culture dishes and cultured with DMEM containing 20% fetal bovine serum overnight. Then, the medium of PTF dishes was added to the rhCTGF protein (R&D Systems) at the concentrations of 0, 50, 100, and 200 ng/ml.

### Immunohistochemistry staining

A total of 101 pairs of human gastric tumor tissues and their non-tumor tissues were collected from the Ruijin Hospital (Shanghai, China). According to the previous description, immunohistochemistry (IHC) was performed (19). The staining intensity was classified into three grades: no staining (1 point), light brown (2 points), and dark brown (3 points). Based on the number of positive cells (percentages), four grades were assigned.: 0%–25% (1 point), 26%–50% (2 points), 51%–75% (3 points), and 76%–100% (4 points). Overall staining score = intensity score × percentage score.

### Real-time quantitative RT-PCR

The cell lines in the logarithmic phase were collected for RNA extraction. Total RNA was extracted utilizing an EZ-press RNA purification kit (EZBioscience), and cDNA synthesis was performed by Reverse Transcription system. The mRNA expression levels were measured using the SYBR Green PCR Master Mix and the Applied Biosystems 7900HT sequence detection system. Gene-specific primers were obtained from Primer-BLAST (US National Library of Medicine) and Primer-Bank (Massachusetts General Hospital, The Center for Computational and Integrative Biology, and Harvard Medical School). The primer sequences were listed in **Table S1**. Glyceraldehyde-3-phosphate dehydrogenase (GADPH) was used as an internal control.

### Western blotting assay

According to the previous description (20), Western blotting assay was performed. Total protein was extracted in radio immunoprecipitation assay (RIPA) lysis buffer (Solarbio Life Sciences) with proteinase inhibitors and phosphatase inhibitors. In order to determine the protein concentration in each lysate, we used a protein assay reagent kit (Thermo Fisher Scientific). Transfection of the proteins onto polyvinylidene difluoride membranes was carried out after electrophoresis (Millipore). Then, the membranes were blocked for 1.5 h in tris-buffered saline (TBS) with 5.0% bovine serum albumin (BSA) and probed with the primary antibodies. After being washed three times with tris buffered saline with tween 20 (TBST), the protein content was incubated with Goat Anti-Rabbit IgG (H+L), Horseradish Peroxidase (HRP) conjugate (Proteintech), or Goat Anti-Mouse IgG (H+L), HRP conjugate. The primary antibodies used were listed below: GAPDH (1:1,000, Proteintech), S100A4 (1:1,000, Abcam), and fibroblast activation protein (FAP) (1:1,000, Abcam).

## Results

### The landscape of the tumor microenvironment in gastric cancer

**Figure 1:**
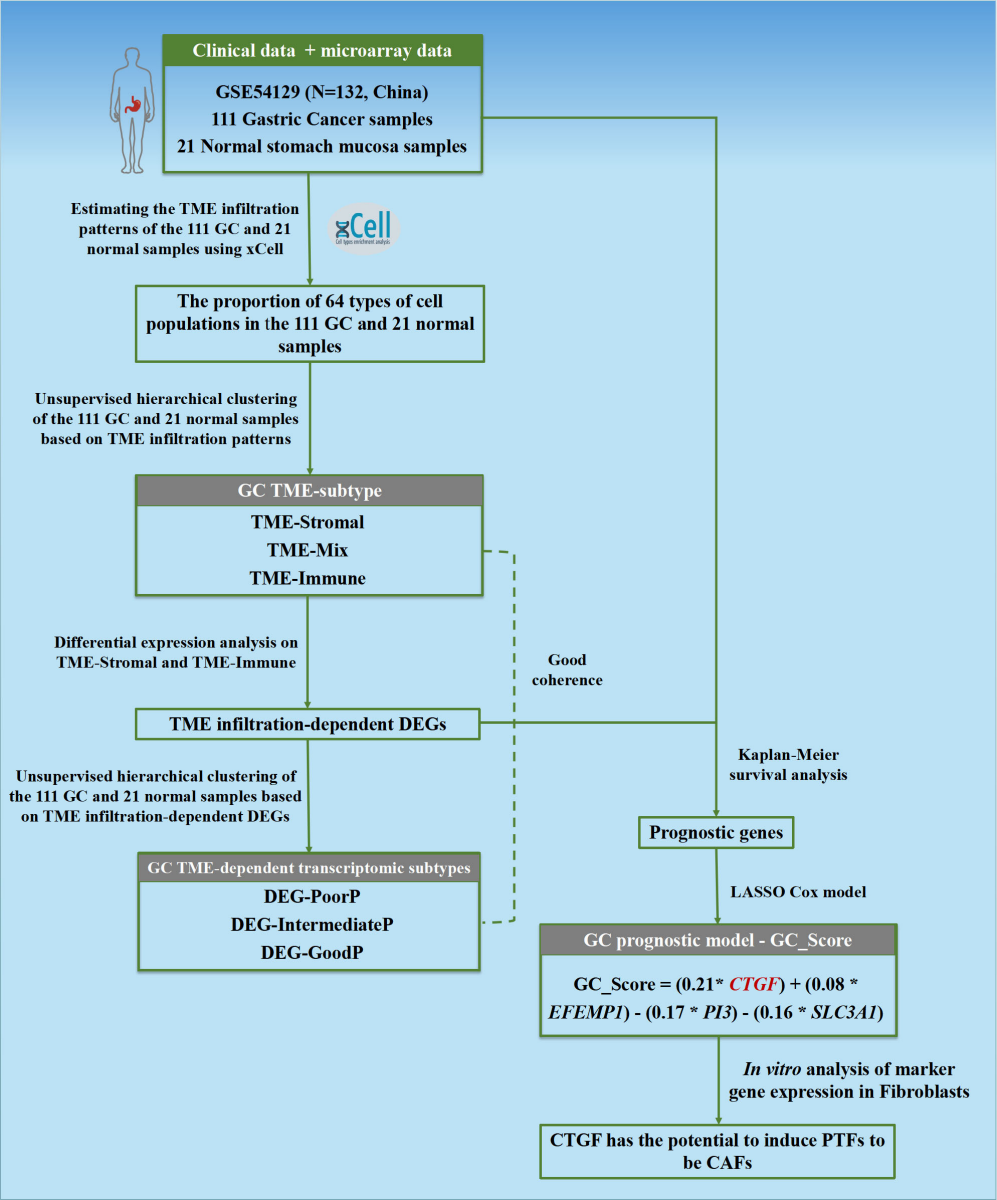
Flowchart of the study. The TME infiltration patterns of 111 GC and 21 normal stomach mucosa samples based on bulk transcriptomic profiles were estimated in this study, and a predictive model GC_Score for prognosis and drug responses with interpretability for carcinogenesis was developed. Furthermore, a TME-modulating gene, CTGF, was proposed to activate CAFs, thereby promoting the progression of GC.

**Figure 2:**
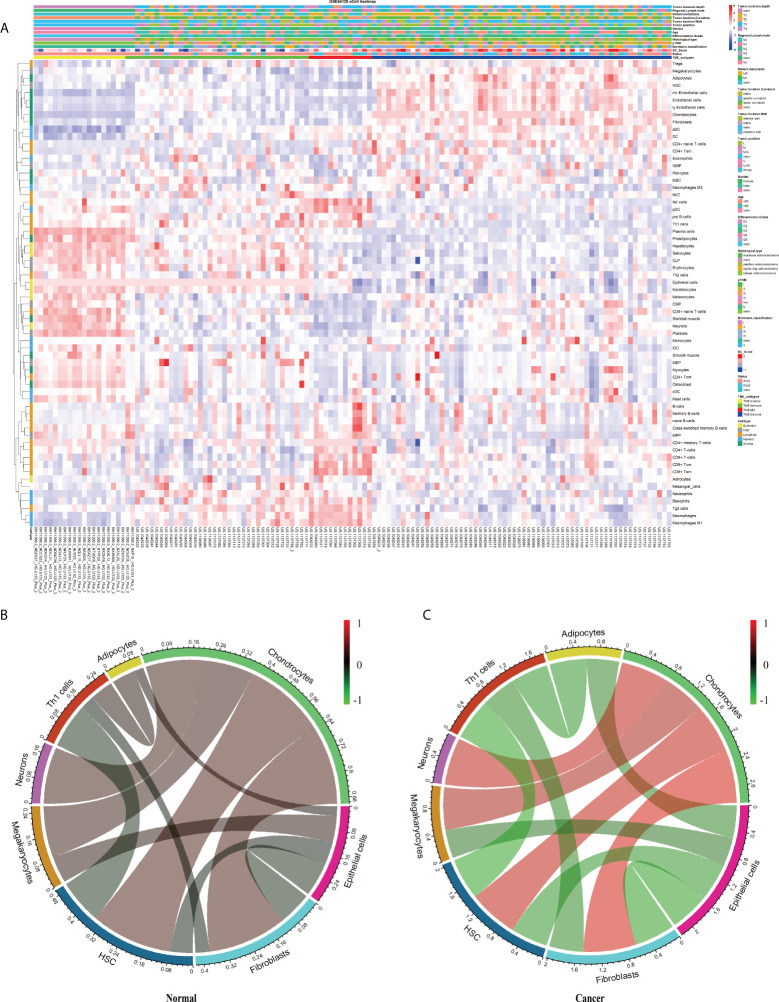
The landscape of the TME in GC. **(A)** Heatmap of 64 TME cells for 111 GC and 21 normal samples. pTNM, Borrmann classification, Tumor invasion depth, Regional lymph node, Distant metastasis, Tumor location, Tumor position, Gender, Age, Tissue differentiation, Histological type, Status, Cluster, and CellType were shown as patient annotations. **(B, C)** Correlation of cell proportion among the 12 survival-related cell populations in normal and tumor groups. Red, positive correlation; green, negative correlation; black, no correlation. The width of the connection line was correlated with the absolute value of its corresponding Spearman correlation coefficient. Note that the cell bar in panels **(B)** and **(C)** has a different scale.

This study’s schematic overview can be seen in **Figure 1**. The TME infiltration landscapes of 111 GC and 21 normal stomach mucosa samples (Ruijin cohort, GSE54129) were characterized by using the xCell tool, and a total of 64 cell types were taken into consideration (**Table S2**). As shown in **Figures 2A**, **S1A**, the normal gastric mucosae were mainly composed of lymphoid and epithelial cells (**Figure S1D**), while the GC samples exhibit higher heterogeneity with more cell types involved, such as stroma and HSC, but with less common cell types across patients compared with normal samples.

**Figure 3:**
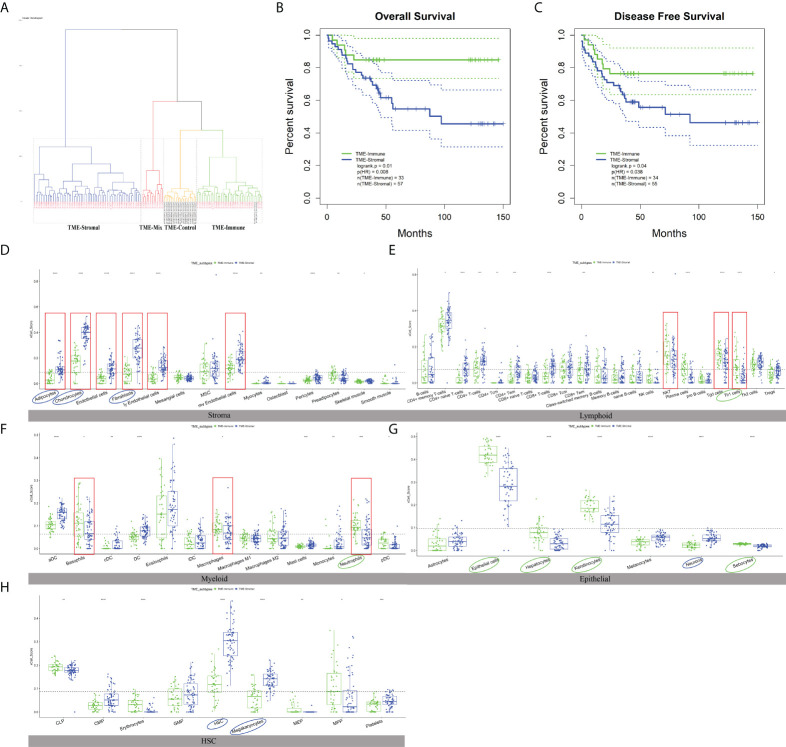
The characteristics of TME subtypes. **(A)** Unsupervised clustering of GSE54129 cohort with matched xCell scores; the samples named in black font was the normal group. **(B, C)** Kaplan–Meier curves for overall survival (OS) and disease-free survival (DFS) of GC patients with the TME subtypes (log-rank test). **(D–H)** The distribution of five cell types included 64 TME cells in TME-Stromal and TME-Immune subtypes. TME-Stromal and TME-Immune were shown in blue and green, respectively. The cell types associated with poor clinical outcomes were circled in blue, while the cell types associated with good clinical outcomes were circled in green. The dominant cell types displayed a significant difference proportion in TME-Stromal and TME-Immune noted by the red rectangular frame. The thick line represented the median value. The bottom and top of the boxes were the 25th and 75th percentiles (interquartile range). The dotted line showed the average score of each cell type. The cells enclosed by the circle were significant for OS. The statistical difference was compared through the t-test. * p < 0.05; ** p < 0.01; *** p < 0.001; **** p < 0.0001.

We then checked the prognosis significance of the abundance of individual cell types. Kaplan–Meier survival analysis identified 12 cell types that were independently associated with OS with p ≤ 0.01 (**Figure S2**, **Figures 3D–H**, noted by circles) and hereinafter referred to as survival-related cell types. Specifically, the TME of the GC patients with poor prognosis was abundantly infiltrated with adipocytes, chondrocytes, fibroblasts, HSC, megakaryocytes, and neurons (**Figures 3D–H**, noted by blue circles) and depleted with epithelial cells, hepatocytes, keratinocytes, neutrophils, sebocytes, and Th1 (**Figures 3D–H**, noted by green circles).

Aiming to explore potential coordination between survival-related cell types, we calculated Spearman correlation coefficients of cell proportion among the above 12 survival-related cell types in normal and tumor tissues. In general, the absolute value of the Spearman correlation coefficient between cell types turned larger from normal to tumor [Cor _normal_ = 0.34 (average); Cor _cancer_ = 0.53 (average)], suggesting a closer communication among cells in tumor (**Figures 2B, C** **)**. Cell interactions with the change of correlation coefficient between normal and tumor groups greater than 0.5 were retained, resulting in a total of 11 pairs of cell types, involving eight cell types, adipocytes, chondrocytes, epithelial cells, fibroblasts, HSC, megakaryocytes, neurons, and Th1 cells (**Figures 2B, C** **;** **Table 1**). Considering fibroblasts play an important role in tumor invasion and metastasis (21), we especially examined cell communication related to fibroblasts. Fibroblasts and chondrocytes, both displaying high proportion in poor-prognosis GC patients (**Figures S2B, C**), had a weak positive correlation in normal (Cor = 0.26) but a strong positive correlation in tumor (Cor = 0.84). Different from fibroblasts–chondrocytes pair, fibroblasts and Th1 cells and fibroblasts and epithelial cells appeared to have no correlations in normal (Cor _Fibroblasts-Th1 cells_ = -0.05, Cor _Fibroblasts-Epithelial cells_ = -0.11) while showing strong negative correlations in tumor (Cor _Fibroblasts-Th1 cells_ = -0.55, Cor _Fibroblasts-Epithelial cells_ = -0.64) (**Table 1**). These observations are accordant with the reports that fibroblasts and chondrocytes have a positive cross-talk during disease progression in skeletal-related diseases (22, 23), that fibroblasts inhibit the proliferation of Th1 cells in rheumatoid arthritis (24), and that uncontrolled continued transition from epithelial cells to fibroblasts through the epithelial–mesenchymal transition (EMT) confers metastasis-initiating abilities (25). Also, it is consistent with our observation that patients with a lower proportion of fibroblasts, a higher proportion of Th1 cells, or a higher proportion of epithelial cells, displayed better survival (**Figures S2L, G**).

**Table 1:** Correlation coefficients between cell types with the change between normal and tumor. greater than 0.5.

Cell_1	Cell_2	Cor (normal)	Cor (tumor)	Abs [Cor (normal) – Cor (tumor)]
**Fibroblasts**	**Chondrocytes**	0.26	0.84	0.58
Neurons	Chondrocytes	0.19	0.76	0.57
HSC	Chondrocytes	0.27	0.79	0.51
Megakaryocytes	Chondrocytes	0.17	0.68	0.51
**Fibroblasts**	**Th1 cells**	-0.05	-0.55	0.5
Th1 cells	HSC	-0.15	-0.66	0.51
**Fibroblasts**	**Epithelial cells**	-0.11	-0.64	0.52
HSC	Epithelial cells	-0.07	-0.59	0.52
Megakaryocytes	Epithelial cells	0.09	-0.45	0.54
Epithelial cells	Adipocytes	0.03	-0.52	0.55
Th1 cells	Adipocytes	0.08	-0.59	0.67

Cell type pairs related to fibroblasts were labeled in bold.

### Cell infiltration-based tumor microenvironment subtypes of gastric cancer

To check the association of the TME infiltration pattern with clinical outcome, unsupervised hierarchical clustering (26) of the 111 GC and 21 normal samples was performed with their matched xCell scores, i.e., the proportions of 64 cell types (distance = “manhattan”, method = “ward.D”). A total of four clusters were obtained (**Figure 3A**), where the normal samples formed a separate cluster, termed “TME-Control.” The three tumor-related clusters were taken as TME subtypes, which showed significantly different OS (log-rank test, p = 0.03) (**Figure S3**), indicating the reliability of TME-based subtyping. Fisher’s exact test indicated that the TME subtypes were significantly correlated with histological type (p = 0.035), Borrmann classification (p = 0.003), and tumor location (p = 0.039) among the clinicopathologic variables (**Table S3**).

In order to check the difference between the TME subtypes in their cell abundance and explore the functional relevance of cell infiltration patterns, we defined dominant cell types in each sample cluster (subtype). First of all, the proportion value of each cell type was ranked among all samples within a cluster, and the median values of the 64 cell types were retrieved. For a specific subtype, the top 20 out of the 64 cell types were taken to determine the dominant cell types of the subtype. In the worst-prognosis subtype (marked in blue in **Figure 3A**), seven out of the top 20 cell types were stromal cells, accounting for 50% of stromal cells, including adipocytes, chondrocytes, endothelial cells, fibroblasts, ly endothelial cells, mv endothelial cells, mesenchymal stem cells (MSCs) (**Figure S1B**), accordant with the roles of stromal cells in tumor invasion and metastasis. The worst-prognosis cluster was therefore termed “TME-Stromal” (**Figures 3B, C** **)**. While in the best-prognosis subtype (marked in green in **Figure 3A**, **Figures 3B, C** **)**, 11 out of the top 20 cell types were immune cells, accounting for 32% of immune cells, including CD4^+^ memory T cells, CD4^+^ T cells, Natural killer T cell (NKT), gamma delta T cells (Tgd cells), Th1 cells, Th2 cells, aDC, basophils, eosinophils, macrophages, and neutrophils (**Figure S1C**), consistent with the tumor inhibitory functions of immune cells. This cluster was then termed “TME-Immune.” **Figures 3B** and **2C** showed the significant differences in OS (log-rank test, p = 0.01) and DFS (log-rank test, p = 0.04) between TME-Stromal and TME-Immune.

It was noticed that the third subtype (marked in yellow in **Figure 3A**) involved even more immune cells (12 out of the top 20 cell types) and the same number of stromal cells (three out of 20) as TME-Immune but displayed worse prognosis than TME-Immune (**Figure S3**). This might be attributed to the contribution of specific cell types instead of simply calculating the number of immune- or stromal-related cell populations. This cluster was then termed “TME-Mix,” and the following study was focused on TME-Stromal and TME-Immune.

As expected, all of the dominant stromal cell types for TME-Stromal except MSCs displayed a significantly higher proportion in TME-Stromal than in TME-Immune (**Figure 3D**, noted by the red rectangular frame). Similarly, among the 11 dominant immune cell types for TME-Immune, NKT, Tgd cells, Th1 cells, basophils, macrophages, and neutrophils had a significantly higher proportion in TME-Immune compared with TME-Stromal (**Figures 3E, F**, noted by the red rectangular frame).

Among the 12 survival-related cell types (noted by circles in **Figures 3D–H**, **Figure S2**), six cell types (adipocytes, chondrocytes, fibroblasts, neurons, HSCs, megakaryocytes) had a higher proportion in TME-Stromal compared with TME-Immune (noted by blue circles in **Figures 3D–H**), and the high proportion was associated with poor clinical outcomes (**Figure S2**). While the other six cell types (Th1 cells, neutrophils, epithelial cells, hepatocytes, keratinocytes, sebocytes) were associated with good clinical outcomes (noted by green circles in **Figures 3D–H**) showed a higher proportion in TME-Immune than in TME-Stromal.

### Identification of tumor microenvironment infiltration-dependent differentially expressed genes

**Figure 4:**
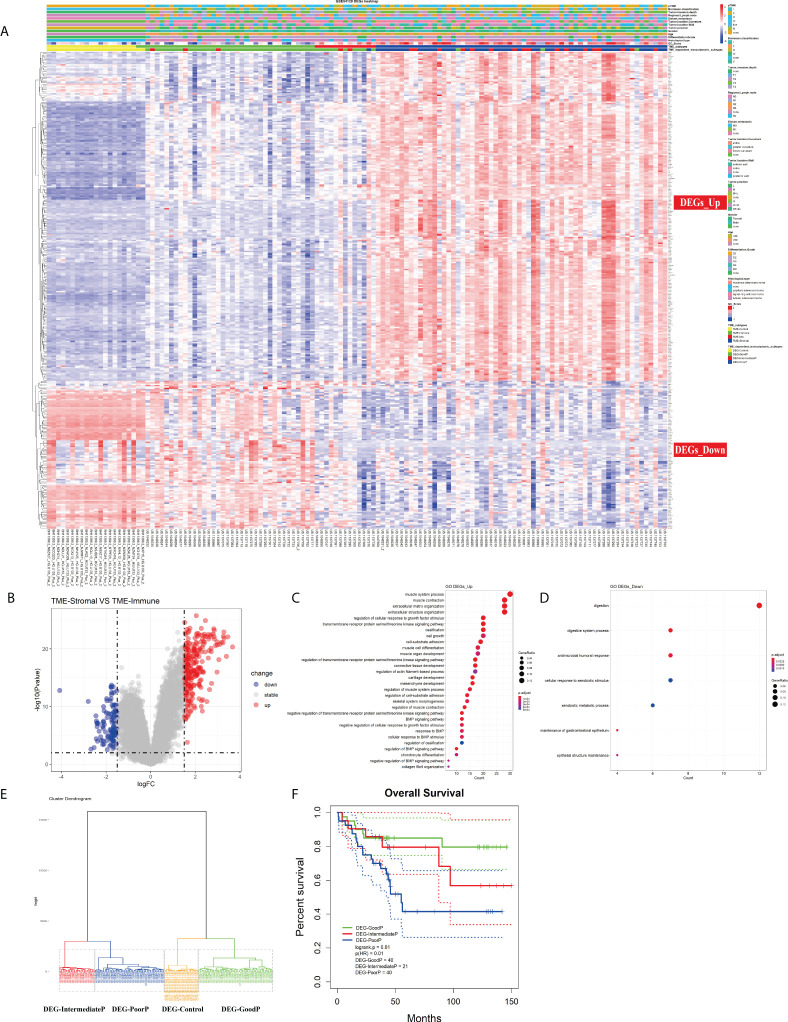
Investigation of the TME infiltration-dependent expression change. **(A)** Heatmap of the 345 differentially expressed genes (DEGs) between TME-Stromal and TME-Immune. **(B)** Volcano plot of the 345 DEGs. **(C, D)** GO enrichment analysis of the 345 DEGs: DEGs_Up and DEGs_Down. **(E)** Unsupervised hierarchical clustering of the 345 DEGs based on expression data to classify patients into four groups: DEG-Control, DEG-GoodP, DEG-IntermediateP, and DEG-PoorP. **(F)** Kaplan–Meier curves for overall survival (OS) of GC patients with the TME-dependent transcriptomic subtypes (log-rank test).

Although TME infiltration patterns have recently been correlated to clinical features in various cancers, the linkage between cellular interactions in the TME and the underlying molecular events remains obscure. To address this issue, we first identified 345 DEGs between TME-Stromal (poor prognosis) and TME-Immune (good prognosis) by using the R package Limma with log2 (fold change) >1.5 and adjusted p < 0.01. Among them, 238 were upregulated in TME-Stromal and 107 were downregulated, which were regarded as relevant to tumor progression, and named DEGs_Up and DEGs_Down, respectively (**Figures 4A, B**; **Table S4**). The potential functions of these two sets of genes were then inferred with functional enrichment analysis by using the R package clusterProfiler. The DEGs_Up genes were enriched in matrix remodeling and cell proliferation-related pathways, such as ECM organization, extracellular structure organization, connective tissue development, mesenchyme development, regulation of cellular response to growth factor stimulus, and cell growth (**Figure 4C**, **Table S5**). Meanwhile, the DEGs_Down genes were mainly enriched in immunity-related pathways, such as antimicrobial humoral response and cellular response to xenobiotic stimulus (**Figure 4D**, **Table S5**). Taken together, these upregulated and downregulated genes could explain the distinct prognosis of the patients in TME-Stromal and TME-Immune.

We then carried out unsupervised hierarchical clustering on the 111 GC and 21 normal samples based on the expression data of the 345 DEGs. Similar to the above TME infiltration-based clustering result, the normal samples formed a separate cluster (DEG-Control), supporting the reliability of the TME-dependent DEG-based clustering. Again, besides DEG-Control cluster, three clusters were obtained and marked as DEG-GoodP, DEG-IntermediateP, and DEG-PoorP (**Figure 4E**) according to their prognosis. The three tumor subtypes showed significant differences in OS (**Figure 4F**), with DEG-PoorP corresponding to the worst prognosis and DEG-GoodP corresponding to the best prognosis (log-rank test, p = 0.01). More importantly, the TME-dependent transcriptomic subtypes were basically consistent with the TME subtypes, which confirmed the linkage between the TME cell infiltration pattern and gene expression pattern, and indicated the functional relevance of the DEGs used in the clustering process in explaining the TME subtypes. As shown in the alluvial diagram (**Figure S4**, **Table S6**), the matching rates of the TME subtypes and the TME-dependent transcriptomic subtypes for TME-Immune vs. DEG-GoodP, TME-Stromal vs. DEG-PoorP, TME-Mix vs. DEG-IntermediateP, and TME-Control vs. DEG-Control were 85.71%, 68.42%, 18.18%, and 100%, respectively.

### The gastric cancer prognostic model-GC_score

**Figure 5:**
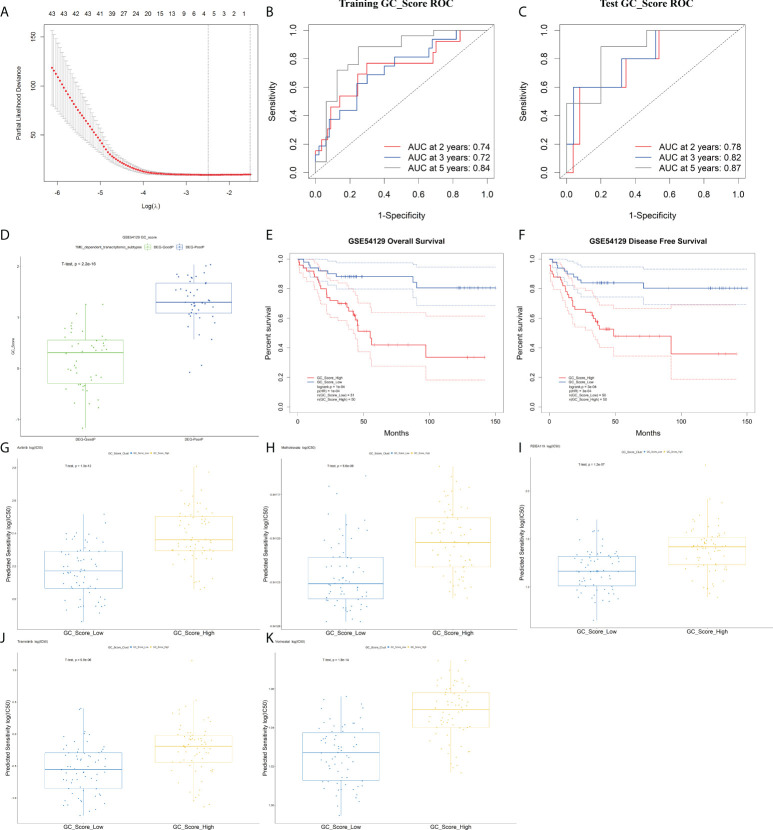
The GC_Score model and its prognostic significance. **(A)** Several cell types were involved in the LASSO model. **(B, C)** The GC_Score was measured by time-dependent receiver operating characteristic (ROC) curves in the training set and the test set. **(D)** The boxplot of GC_Score in DEG-GoodP and DEG-PoorP subtypes. The thick line represented the median value. The bottom and top of the boxes were the 25th and 75th percentiles (interquartile range). **(E, F)** Survival impact of the GC_Score, Kaplan–Meier curves for overall survival (OS) and disease-free survival (DFS) in the GSE54129 cohort. **(G–K)** The boxplot of drug sensitivity in the GC_Score_Low and the GC_Score_High group. **(G)** Axitinib, **(H)** methotrexate, **(I)** RDEA119, **(J)** trametinib, **(K)** vorinostat.

Among the above 345 DEGs between TME-Stromal and TME-Immune, 94 genes were proven to be significantly associated with OS and DFS by Kaplan–Meier survival analysis (**Table S7**). Based on the 94 prognostic genes, we built a GC_Score model with LASSO Cox regression (**Figure 5A**) using the following formula: GC_Score = (0.21* CTGF) + (0.08 * EFEMP1) - (0.17 * PI3) - (0.16 * SLC3A1), which involved four genes (see **Table S8** for their functional annotation, relation with cell types, survival relevance, and so on). The prognostic accuracy of the GC_Score model was investigated with time-dependent ROC analysis. The average Area Under Curve (AUC) values of 2-, 3-, and 5-year prognosis predictions in the training set reached 0.74, 0.72, and 0.84, and the average AUC values of survival predictions in the test set were 0.78, 0.82, and 0.87 (**Figures 5B, C** **)**, respectively. The mean GC_Score of the DEG-PoorP group was higher than that of the DEG-GoodP group (**Figure 5D**). Similarly, the mean GC_Score of TME-Stromal was higher than that of TME-Immune (**Figure S5A**).

The samples were then classified into GC_Score_High and GC_Score_Low groups according to the median value. As expected, Kaplan–Meier survival analyses (**Figures 5E, F** **)** showed that the GC_Score_High group had a poorer OS and DFS compared with the GC_Score_Low group (**Figure S5G**). Moreover, whether in the early or late stages of pTNM, the GC_Score can accurately discriminate the survival of GC patients within the same TNM stages (**Figure S5B, C**).

Furthermore, we predicted the 111 GC patients’ clinical outcomes of chemotherapy based on the tumor gene expression data in GSE54129 by using R packages pRRopheticin that adopted gene expression and drug sensitivity data in a large panel of cancer cell lines (18). It was found that for several commonly used chemotherapeutics in GC like axitinib, RDEA119, methotrexate, trametinib, and vorinostat, the GC_Score_Low group had a lower IC50 value, or higher drug sensitivity, than that of the GC_Score_High group, suggesting that the GC_Score model is also capable of predicting the chemotherapeutic response of patients (**Figures 5G–K**).

To test whether the GC_Score model has robust prognostic value across different populations, the performance of the GC_Score was assessed on three GC cohorts, including two independent Gene Expression Omnibus (GEO) datasets (ACRG, N = 300; GSE15459, N = 191, Singapore) and TCGA-STAD dataset (N = 417). For the three validation cohorts, the GC_Score_High group consistently had a worse OS than that of the GC_Score_Low group (**Figures S5D–F**, log-rank p-values: ACRG = 0.01, GSE15459 = 0.003, TCGA-STAD = 0.03). Collectively, the GC_Score can be used as a robust prognostic signature for GC.

### Connective tissue growth factor has the potential to induce peritumoral fibroblasts to become cancer-associated fibroblasts

**Figure 6:**
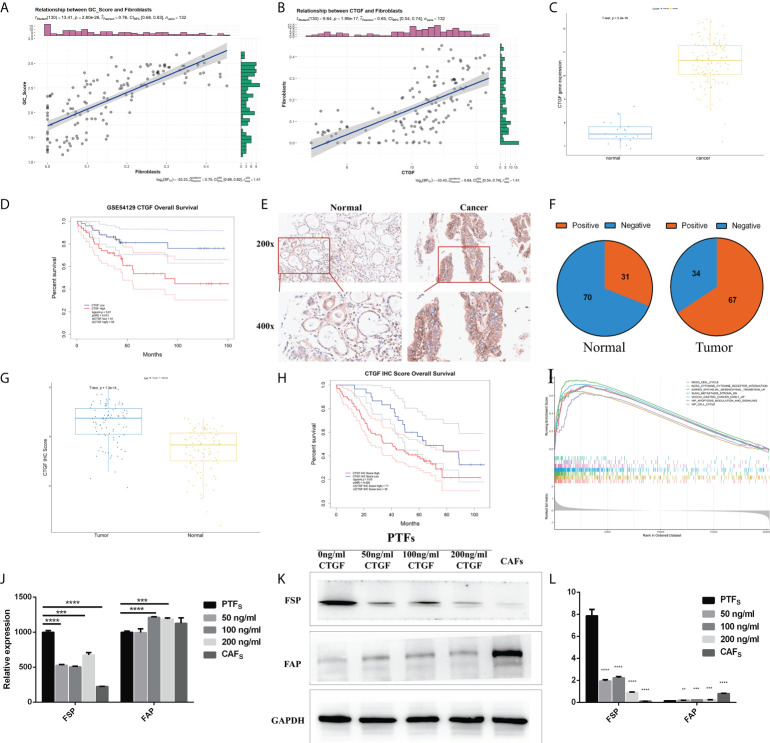
CTGF has the potential to induce PTFs to be CAFs. **(A, B)** Correlations between GC_Score and fibroblasts/CTGF and fibroblasts. The regression lines and confidence interval shadows were calculated by single-variable regression. **(C)** The boxplot of the CTGF expression in normal and cancer samples. The thick line represented the median value. The bottom and top of the boxes were the 25th and 75th percentiles (interquartile range). **(D)** Survival impact of the CTGF expression, Kaplan–Meier curves for overall survival (OS) in the GSE54129 cohort. **(E)** Representative IHC staining with CTGF antibody in GC and paired adjacent non-tumor tissues. Magnification ×200 and ×400. **(F)** The pie graph of IHC Score of CTGF in GC tissues and corresponding non-tumor tissues, Positive: IHC ≥8, Negative: IHC <8. **(G)** The boxplot of IHC Score of CTGF in normal and cancer samples. The thick line represented the median value. The bottom and top of the boxes were the 25th and 75th percentiles (interquartile range). **(H)** Survival impact of IHC Score of CTGF, Kaplan–Meier curves for OS. **(I)** Gene set enrichment analysis (GSEA) of CTGF. **(J)** qRT-PCR detection of FSP and FAP mRNA levels in PTFs, CAFs, and PTFs treated with different concentrations of rhCTGF protein for 50, 100, and 200 ng/ml. **(K, L)** Protein levels of FSP and FAP in PTFs, CAFs, and PTFs treated with different concentrations of rhCTGF protein were analyzed by Western blotting. These data were presented as the mean ± SD; n = 3 independent experiments.

To elucidate the biological relevance of our GC_Score from the viewpoint of TME infiltration, we further calculated the correlations between the GC_Score and each of the 64 TME cell types based on the GSE54129 dataset, and chondrocytes, fibroblasts, and ly endothelial cells held the top 3 (**Figure S6A**). Again, given the important roles of fibroblasts in tumor invasion and metastasis (21), we set out to focus on fibroblasts (**Figure 6A**, r = 0.76, p = 2.8e^-26^). Considering that CTGF occupied the highest weight among the four genes involved in the GC_Score model, we specifically calculated the Pearson correlation coefficient between fibroblasts and CTGF. It was revealed that the expression of CTGF was strongly positively correlated with the proportion of fibroblasts (**Figure 6B**, r = 0.65, p = 1.99e^-17^).

In the GSE54129 cohort, CTGF expression was significantly increased in GC (**Figure 6C**), and the high expression of CTGF was associated with poor OS (**Figure 6D**, log-rank p = 0.01), also in TCGA-STAD (**Figures S6B, C**). We then examined CTGF protein expression in 101 other independent pairs of GC and adjacent non-tumor tissues by IHC (**Figure 6E**). It was found that the positive rate of CTGF was about 66% in GC tissues, while it was approximately 31% in adjacent non-tumor tissues (**Figure 6F**), indicating increased expression of CTGF protein in GC (**Figure 6G**). Furthermore, Kaplan–Meier analysis showed that CTGF expression was inversely correlated to OS in the 101 GC samples (**Figure 6H**). Gene set enrichment analysis (GSEA) was then performed on CTGF-High and CTGF-Low groups (grouped by median) in GSE54129 cohort by using R packages GSVA and clusterProfiler. Based on C2: Kyoto Encyclopedia of Genes and Genomes (KEGG), the differential expression was found to be associated with the EMT, metastasis stroma, gastric cancer early up, lung fibrosis, and so on (**Figure 6I**; **Table S9**).

It was noticed that CTGF was a marker gene for mesangial cells (**Table S8**) (7), while the proportions of mesangial cells were not significantly different between TME-Stromal and TME-Immune [TME-Immune = 0.047 (median), TME-Stromal = 0.041(median), **Table S2**]. We, therefore, speculated that the elevated CTGF expression mainly originated from cancer cells.

Increasing evidence in the past few years has demonstrated that CAFs promote carcinogenesis by maintaining a tumor-supportive and immunosuppressive TME (27). Considering the strong positive correlation between fibroblast abundance and CTGF expression in cancer cells and the inverse correlation between CTGF expression and survival, we conceived the hypothesis that CTGF may have the potential to induce transdifferentiation of PTFs (resting fibroblasts) to CAFs and, in turn, promote cancer progression. We added recombinant CTGF protein (rhCTGF) to the culture medium of PTFs and measured the changes of marker genes/proteins of PTFs. It has been well established that fibroblast-specific protein (FSP) and fibroblast activation protein (FAP) are widely expressed in fibroblasts (28, 29), while PTFs hold the higher expression of FSP and lower expression of FAP, and CAFs display the opposite features (30). As shown in **Figures 6J–L**, after PTFs were treated with rhCTGF (50–200 ng/ml), FSP expression was downregulated and FAP expression was upregulated both in mRNA and protein levels basically in a concentration-dependent way compared with the untreated PTFs. Specifically, although there was no significant difference in FAP transcription level between PTF incubated with or without 50 ng/ml rhCTGF, when the concentration reached 100 and 200 ng/ml of rhCTGF, the mRNA expression of FAP was upregulated, even higher than that in CAFs (**Figure 6J**). Taken together, CTGF seems to have the potential to induce PTFs to be CAFs.

## Discussion

GC is highly heterogeneous in terms of clinical manifestations, therapeutic outcomes, histological morphology, and TME infiltration (31). The TME that refers to the internal and external environment of tumor cells, including not only the structure, function, and metabolism of tumor tissues but also the internal environment of tumor cells themselves, has been proven to have important clinical and pathological significance in predicting prognosis and curative effects (25, 32). Exploring the composition of the TME, the functional relevance of cellular interactions, and the underlying molecular events is critical to understand tumor heterogeneity and develop novel therapeutic treatments (33).

The single-cell RNA sequencing (scRNA-seq) technology is becoming widely used to systematically delineate cellular and molecular heterogeneity in tumors. A recent study (34) reported a single-cell transcriptional atlas of GC from nine tumors and three non-tumor samples (>20,000 cells) and obtained differentiation degree-related subtypes that corresponded well to prognosis and histopathological features of Lauren’s subtypes. Kumar etal. (35) described a more comprehensive single-cell atlas of GC from 31 patients (>200,000 cells) and identified 34 distinct cell-lineage states, some of which exhibited distinct cancer-associated expression profiles. Another very recent study (36) profiled 36,897 cells from eight patients with GC using scRNA-seq, aimed to study the heterogeneity of TME cells in GC. They mainly discussed CAFs in GC TME and revealed the unique roles of CAFs in regulating different aspects of the biology of the TME, including immune modulation, invasion, migration, and angiogenesis. However, considering the discrepancy in single-cell dissociation efficiency, high dropouts, high cost, and naturally low coverage of inter-tumor heterogeneity, scRNA-seq still has obstacles in practical application (37, 38), while large cohorts with bulk transcriptomic data and clinical phenotype information through a long-term follow-up provide valuable and more accessible opportunities to decipher TME infiltration with the assistance of tools designed for estimating the abundance of various cell types based on bulk transcriptome.

In the present study, we applied xCell (7) that integrates ssGSEA and deconvolution approaches to the bulk transcriptomic profile (GSE54129) of 111 GC and 21 normal stomach mucosa samples with matched clinical information and calculated the proportion of 64 types of cell populations in the TME (**Table S2**). Among them, 12 cell types were identified as associated with survival (**Figure S2**, **Figures 3D–H**, noted by circles). Given the significant role of fibroblasts in tumor invasion and metastasis (21), we put a particular focus on fibroblasts and the cell types that had differential correlations between normal and cancer. It was found that fibroblasts had a weak positive correlation with chondrocytes in normal (Cor = 0.26), which turned strongly positive in tumor (Cor = 0.84); meanwhile, fibroblasts had no correlations with Th1 cells and epithelial cells in normal (Cor _Fibroblasts-Th1 cells_ = -0.05, Cor _Fibroblasts-Epithelial cells_ = -0.11), while fibroblasts became strongly negatively correlated with them in tumor (Cor _Fibroblasts-Th1 cells_ = -0.55, Cor _Fibroblasts-Epithelial cells_ = -0.64) (**Figures 2B, C** **;** **Table 1**). Fibroblasts were reported to promote the transdifferentiation of chondrocytes in skeletal-related diseases that could, in turn, stimulate fibroblasts to release proangiogenic factors (22, 23). In rheumatoid arthritis, fibroblasts suppressed the proliferation of Th1 cells by tryptophan metabolism and therefore decreased the secretion of Interferon γ (IFN-γ) in a cell contact-independent manner (24). In the process of EMT, epithelial cells undergo a phenotypic switch through acquiring fibroblast-like properties to exhibit reduced cell–cell adhesion and increased motility, which has been regarded as a driving event in the pathogenesis of cancer, including GC (25, 39). Obviously, the detailed mechanisms underlying the coordination between fibroblasts and chondrocytes, the inhibition of Th1 cells by fibroblasts, and the transition from epithelial cells to fibroblasts during gastric carcinogenesis are worthy of further investigation. It has been well established that fibroblasts synthesize the ECM proteins to maintain the structural integrity of most tissues (40). In line with this, CAFs maintain a tumor-supportive microenvironment by producing components of the ECM, matrix remodeling enzymes, and protumor and proangiogenic cytokines (27). Moreover, CAFs could modulate the immune system, yielding an immunosuppressive TME (21). Clinical and epidemiological studies have demonstrated a strong association between CAFs and poor prognosis in GC (41). The very recent single-cell transcriptional atlas of GC also reported the accrual of CAF subpopulations (35). In our previous work, CAFs can promote EMT and metastasis of GC by secreting hepatocyte growth factor (HGF) (42), Interleukin (IL)-6 (43), and IL-33 (44).

As described above, although the TME landscapes of the 111 GC and 21 normal samples involved a total of 64 cell types, the subsequent analyses were limited to12 survival-related cell types and further focused on fibroblasts. We also obtained the abundance of the other more than 50 cell types. Since NK cells are the first line of defense against transformed and infected cells, we take NK cells as an example. It is well known that the function of NK cells is finely regulated by a balance between signals received through stimulatory and inhibitory receptors (45). Compared with TME-Immune, the inhibitory ligands of NK cells such as CD48, HLA-E, and CLEC2D were highly expressed in TME-Stromal (**Figures S7A–C**). Specially, the correlation between CLEC2D and its receptor KLRB1 (46) was lower in TME-Immune (cor = 0.38, p = 0.02) than in TME-Stromal (cor = 0.6, p = 3.09e-07) (**Figures S7D, E**). It is indicated that the overexpression of inhibitory ligands of NK cells may cause immunosuppression and lead to worse outcomes in GC patients.

As shown in **Figures 3B, C** and **Figure S3**, the three GC TME subtypes based on the proportions of 64 cell types displayed distinct survival outcomes. A GC prognostic model was then built based on the DEGs between TME-Stromal (poor prognosis) and TME-Immune (good prognosis) (**Figure 5A**). Quite encouragingly, the GC_Score could accurately discriminate the survival of patients within the same TNM stages (**Figures S5B, C** **)**; moreover, the GC_Score model is capable of predicting the chemotherapeutic response of patients to several common chemotherapeutic drugs for GC (**Figures 5G–K**). As the clinical translation of molecular targets of GC has been disappointing, our study aimed to discover novel genes correlated with drug response in GC, which may be a hint for further investigation on therapeutic mechanisms of these drugs. We also found some studies that supported our results. Sun etal. (47) reported that CTGF increased the sensitivity of rapidly accelerated fibrosarcoma isoform B inhibitor (BRAFi)-resistant cells to vemurafenib (BRAF inhibitor). It is acknowledged that MEK is the downstream molecule of BRAF/mitogen-activated extracellular signal-regulated kinase (MEK)/extracellular signal regulatedkinase (ERK) pathway, and RDEA119 is the MEK inhibitor. So CTGF might participate in GC sensitivity of RDEA119 *via* the BRAF/MEK/ERK pathway. Also, Hua etal. (48) found that HDAC7 participated in CTGF production and cell fibrosis. As CTGF is the downstream target of Histone Deacetylase 7 (HDAC), it might participate in GC response to HDAC inhibitor methotrexate. As expected, fibroblasts held almost top correlation to GC_Score among the 64 cell types (**Figure 6A**, **Figure S6**, r = 0.76, p = 2.8e^-26^). We believe that a combination of our GC_Score and the conventional clinicopathological characteristics will allow better prediction of prognosis and drug responses.

Beyond identifying transcriptome features of certain subtypes as scRNA-seq studies always did, we explored molecular events underlying fibroblast infiltration during carcinogenesis. First, we analyzed the correlation between fibroblasts and CTGF (**Figure 6B**), the highest-weighted gene in our GC_Score model. Interestingly, in the RNA single cell-type data of THE HUMAN PROTEIN ATLAS database, we also see that CTGF has a high correlation with fibroblasts. By examining marker gene expression at RNA and protein levels, we have proven that CTGF has the potential to induce transdifferentiation of PTFs to CAFs (**Figures 6J–L**). It has been established that contact between cancer cells and fibroblasts can promote the CAF phenotype in cancer through a variety of signaling pathways, such as Notch signaling, inflammatory signaling, Janus kinase-signal transducer and activator of transcription (JAK-STAT) signaling, and Yes-associated protein/Transcriptional enhanced associate domain (YAP1/TEAD) signaling (21). Particularly, CTGF was reported to be among the genes associated with CAFs (21). However, the tissue-specific and context-specific mechanisms remain to be demonstrated. The *in vitro* experimental data (**Figures 6J–L**), in combination with our computational results, indicated that highly expressed CTGF from GC cells is capable of promoting the transdifferentiation of PTFs to CAFs. In this sense, CTGF could be regarded as a TME-modulating gene in GC and thus a potential therapeutic target for CAF-targeted therapy. It is worth noting that compared with Transforming growth factor-β (TGF-β) (49), which is an inducer of CTGF expression, CTGF mediates fibrosis and protumor effects but does not participate in anti-inflammatory and antitumor effects (50) and therefore might be more promising for antitumor drug development than TGF-β. So far, there is a line of CTGF-targeted drugs in clinical trials (51, 52), most of which are designed for treating fibrosis-related diseases, while ocaperidone and pamrevlumab also include pancreatic cancer as their indications (53). Here we propose that CTGF-targeted drugs might be repositioned to control gastric carcinogenesis.

## Conclusion

The present work characterizes a comprehensive TME landscape of GC involving 64 cell populations and develops a predictive model GC_Score for prognosis and drug responses with interpretability for carcinogenesis. Furthermore, a TME-modulating gene, CTGF, was proposed to activate CAFs, thereby promoting the progression of GC. This work provides a feasible framework for exploring molecular events underlying TME cell infiltration based on bulk-sequencing data, which makes a complement to scRNA-seq based methodologies. By linking TME cell infiltration and molecular features, it proved useful to interpret carcinogenesis and proposes novel strategies for GC treatment.

## Data availability statement

The original contributions presented in the study are included in the article/**Supplementary Material**. Further inquiries can be directed to the corresponding authors.

## Ethics statement

The studies involving human participants were reviewed and approved by Ruijin Hospital Ethics Committee, Shanghai Jiao Tong University School of Medicine. The patients/participants provided their written informed consent to participate in this study.

## Author contributions

BL and Y-YL conceived the project and supervised the study. QS, WD, YC, JXL, and FL performed the data analysis. JY, JL, BY, and HF conducted basic molecular experiments. ZF contributed to prognostic information collection. XW and JH contributed to the biopsies collection. Z-GZ and LS provided methodology development. QS and Y-YL wrote the manuscript. All authors contributed to the article and approved the submitted version.

## Funding

This work was supported by the National Natural Science Foundation of China (No. 81772509 and 82072605 to BL, No. 81871904 to Z-GZ, No. 82003169 to HR Feng, No. 81871902 and No. 82072616 to LS, No. 32000472 to WD, No. 81902393 to BY, and No. 32100531 to YC), and Natural Science Foundation of Shanghai (No. 19ZR1431700 to BY).

## Conflict of interest

The authors declare that the research was conducted in the absence of any commercial or financial relationships that could be construed as a potential conflict of interest.

## Publisher’s note

All claims expressed in this article are solely those of the authors and do not necessarily represent those of their affiliated organizations, or those of the publisher, the editors and the reviewers. Any product that may be evaluated in this article, or claim that may be made by its manufacturer, is not guaranteed or endorsed by the publisher.

## Supplementary material

Supplementary Figure 1 Cell types in TME **(A-C)** The boxplot of 64 cell types in TME-Control, TME-Stomal, and TME-Immune subtypes. **(D)** The boxplot of five Types in 21 normal samples.

Supplementary Figure 2 Survival impact of the 12 genes significant for survival, Kaplan-Meier curves for overall survival (OS) in the GSE54129 cohort.

Supplementary Figure 3 Kaplan-Meier curves for overall survival (OS) of GC patients with the TME subtypes (log-rank test).

Supplementary Figure 4 Alluvial diagram showing differences among patients by TME subtypes, TME-dependent transcriptomic subtypes, CG_Score, and Survival outcome.

Supplementary Figure 5 The GC_Score model and its prognostic significance. **(A)** The boxplot of GC_Score in TME-Stomal and TME-Immune subtypes. The thick line represented the median value. The bottom and top of the boxes were the 25th and 75th percentiles (interquartile range). **(B, C)** Survival impact of the GC_Score, Kaplan-Meier curves for overall survival (OS) and disease-free survival (DFS) in the GSE54129 cohort. **(D–F)** Survival impact of the GC_Score, Kaplan-Meier curves for overall survival (OS) in the ACRG, GSE15459, and TCGA-STAD cohorts. **(G)** Forest plot of hazard ratios from multivariable Cox proportional hazard regression mode.

Supplementary Figure 6
**(A)** Histogram of correlation between 64 cell types and the GC_Score. **(B)** The boxplot of CTGF in TCGA-STAD cohort. **(C)** Survival impact of CTGF, Kaplan-Meier curves for overall survival (OS) in the TCGA-STAD cohort.

Supplementary Figure 7 The inhibitory receptors of NK cells **(A-C)** The boxplot of inhibitory receptors of NK cells. **(D, E)** Histogram of correlation between inhibitory receptors and ligands of NK cells in TME-Stomal and TME-Immune subtypes.
