# Supplementary figures and images for: Identification of prognostic gene expression signatures based on the tumor microenvironment characterization of gastric cancer
Source: Front Immunol. 2022 Aug 12;13:983632. doi: 10.3389/fimmu.2022.983632 (PMC9411533; doi:10.3389/fimmu.2022.983632)

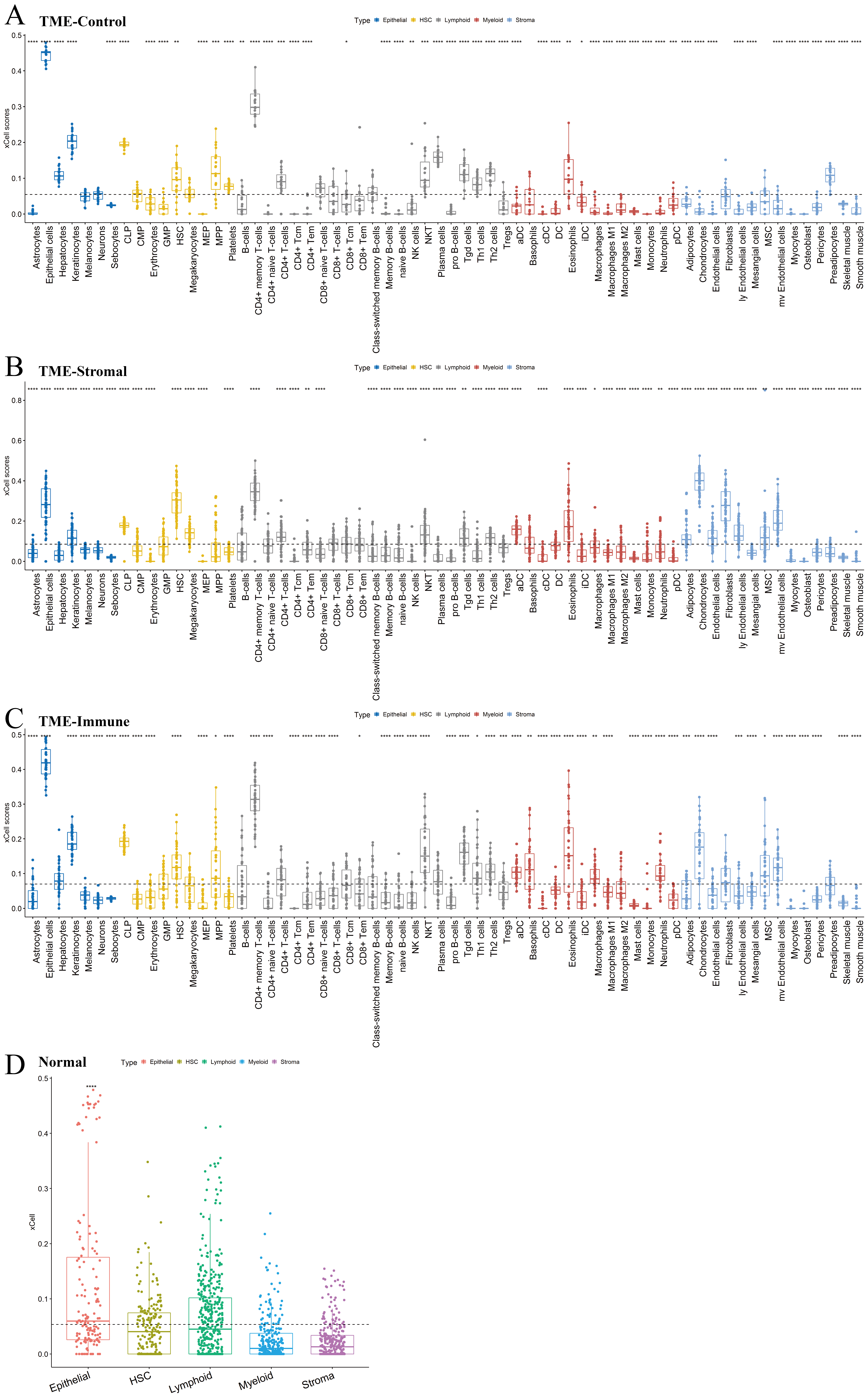

Supplement: Supplementary Figure 1 — Cell types in TME (A-C) The boxplot of 64 cell types in TME-Control, TME-Stomal, and TME-Immune subtypes. (D) The boxplot of five Types in 21 normal samples. [file Image_1.tif]

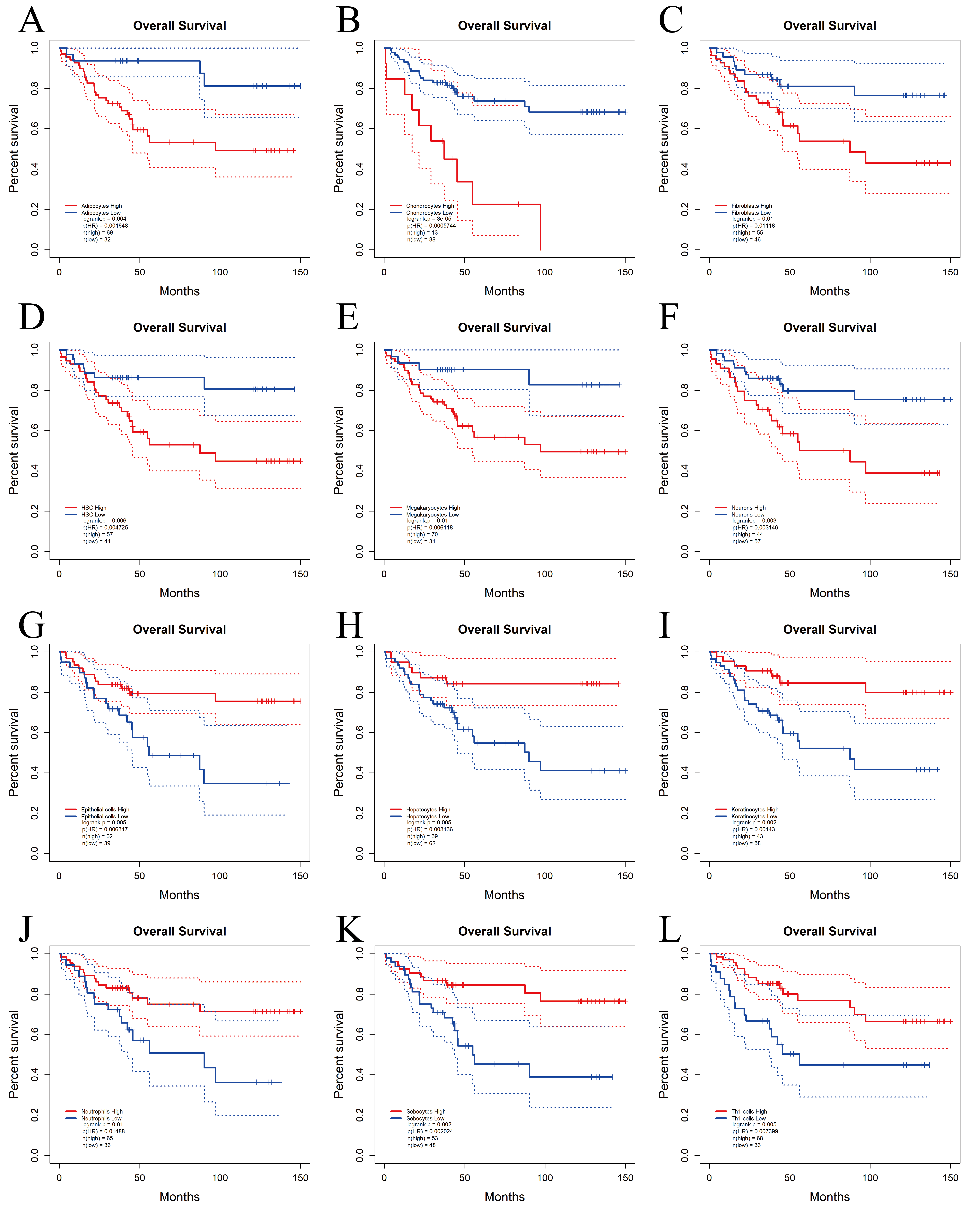

Supplement: Supplementary Figure 2 — Survival impact of the 12 genes significant for survival, Kaplan-Meier curves for overall survival (OS) in the GSE54129 cohort. [file Image_2.tif]

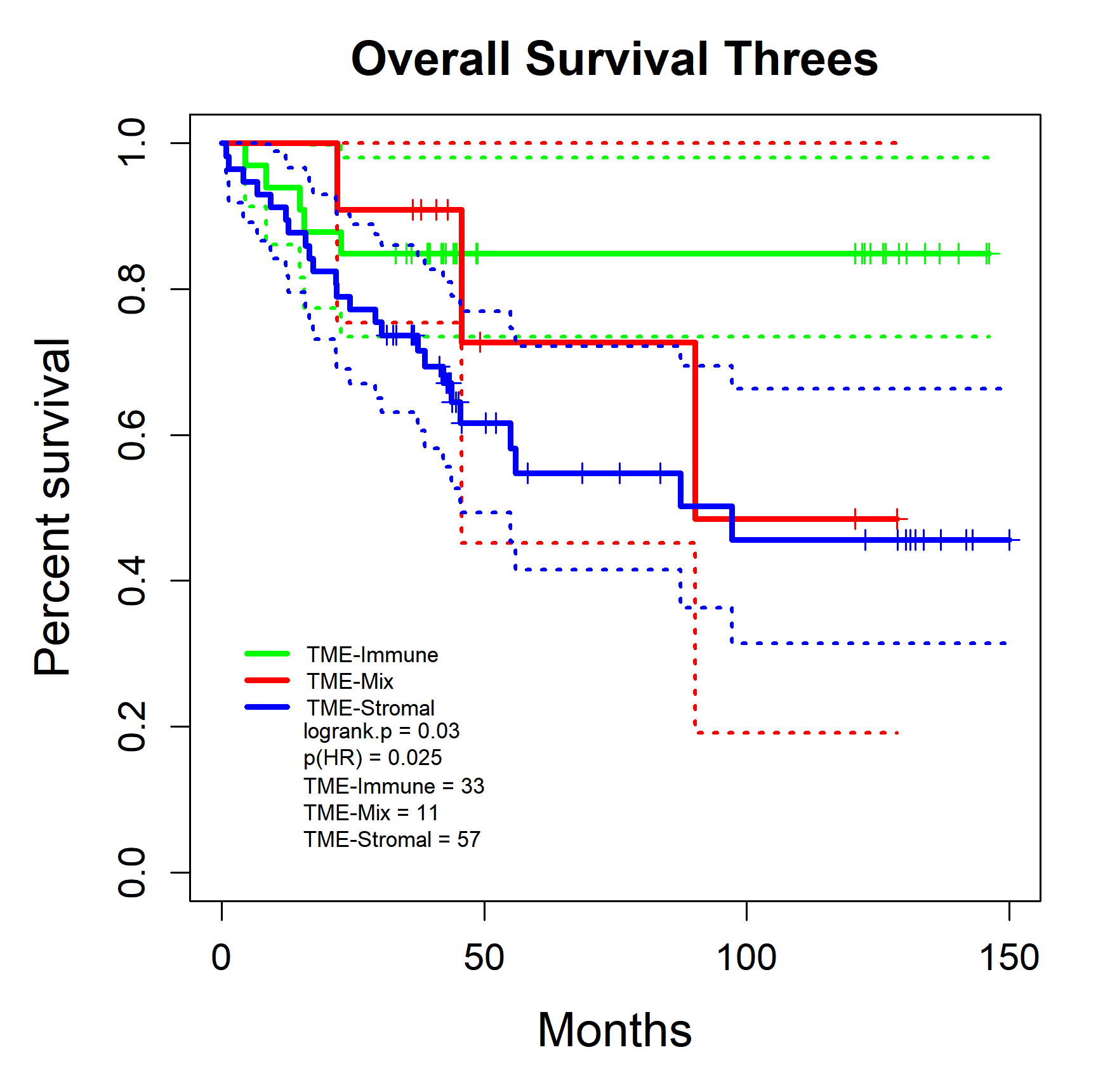

Supplement: Supplementary Figure 3 — Kaplan-Meier curves for overall survival (OS) of GC patients with the TME subtypes (log-rank test). [file Image_3.tiff]

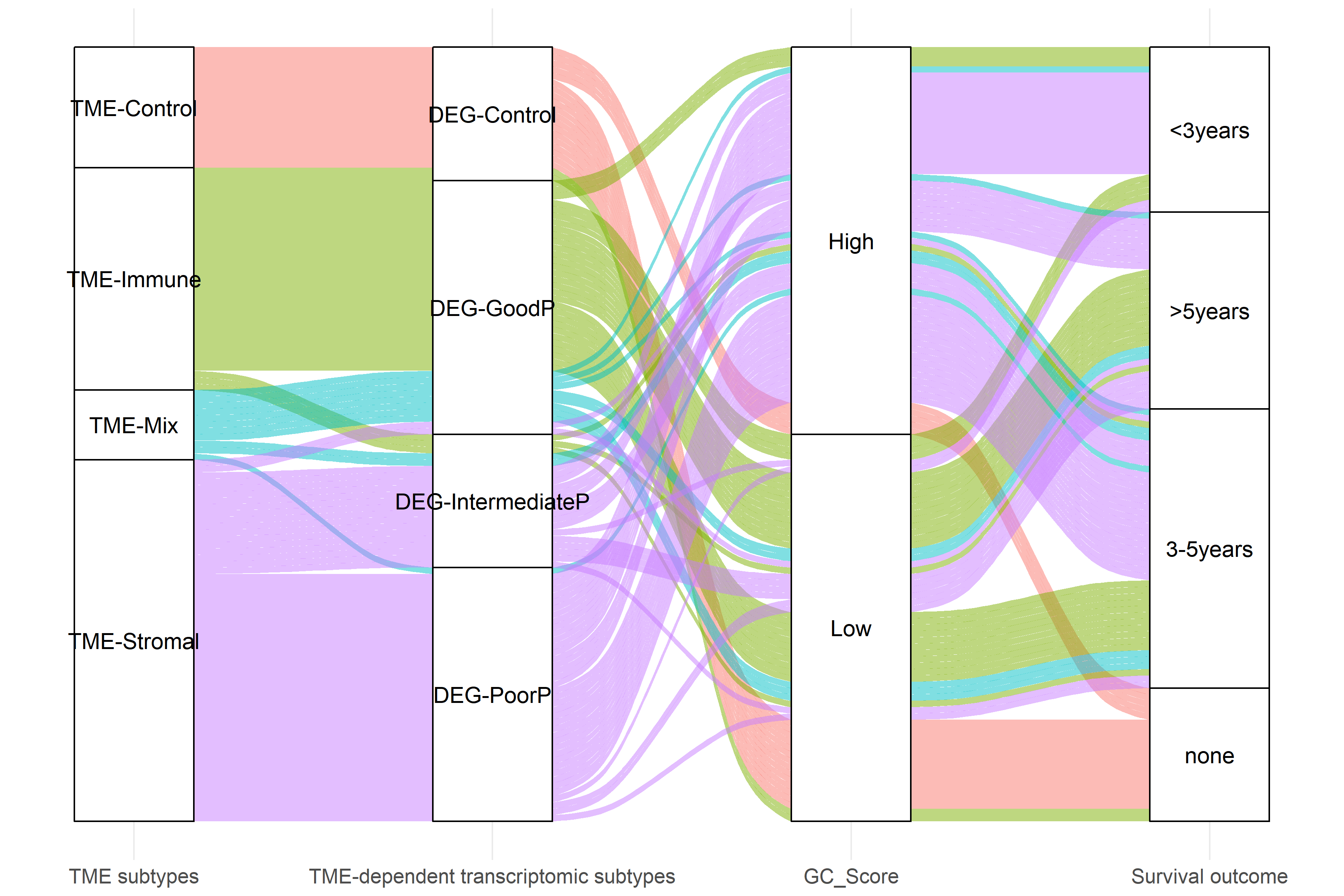

Supplement: Supplementary Figure 4 — Alluvial diagram showing differences among patients by TME subtypes, TME-dependent transcriptomic subtypes, CG_Score, and Survival outcome. [file Image_4.tiff]

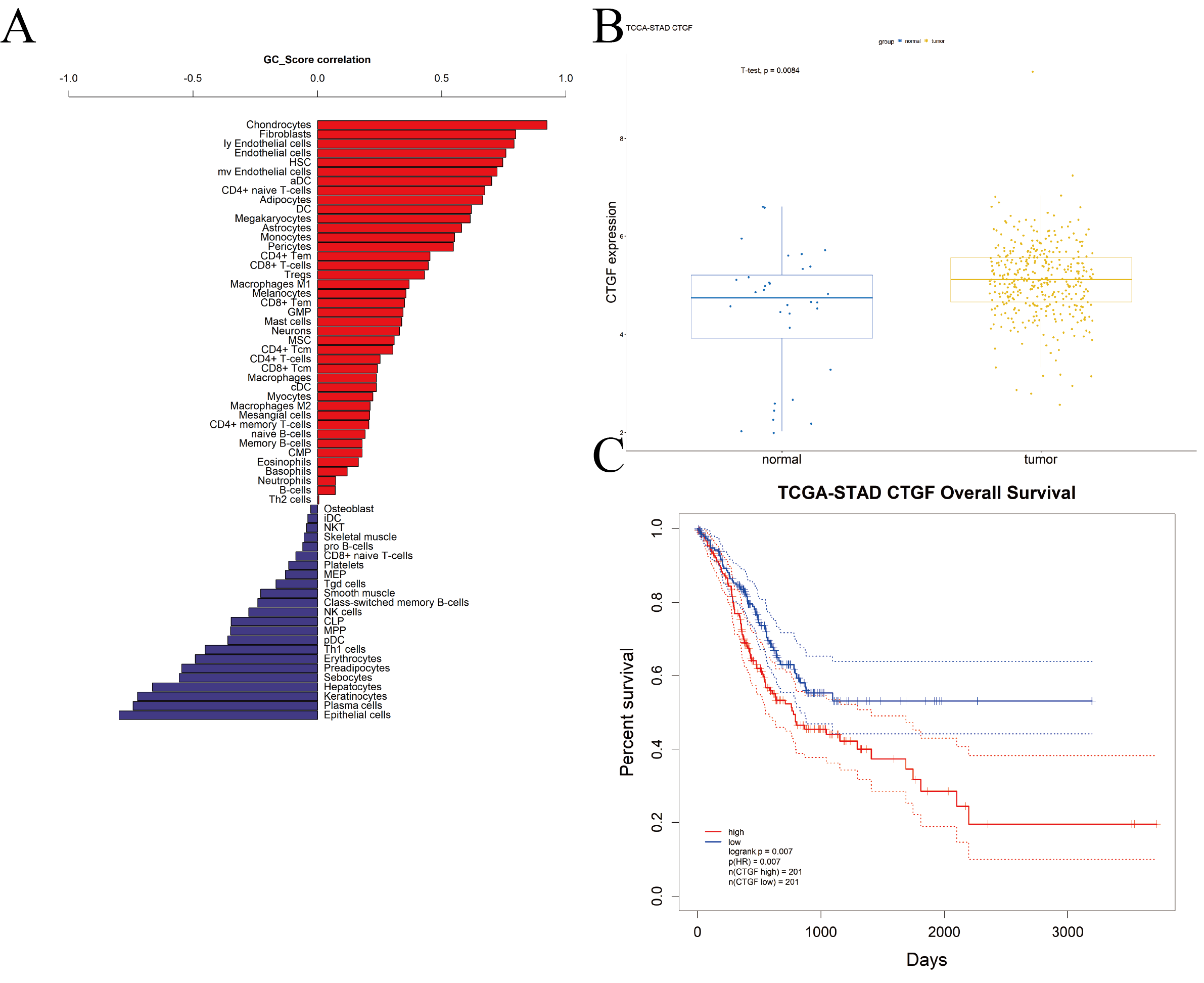

Supplement: Supplementary Figure 6 — (A) Histogram of correlation between 64 cell types and the GC_Score. (B) The boxplot of CTGF in TCGA-STAD cohort. (C) Survival impact of CTGF, Kaplan-Meier curves for overall survival (OS) in the TCGA-STAD cohort. [file Image_6.tif]

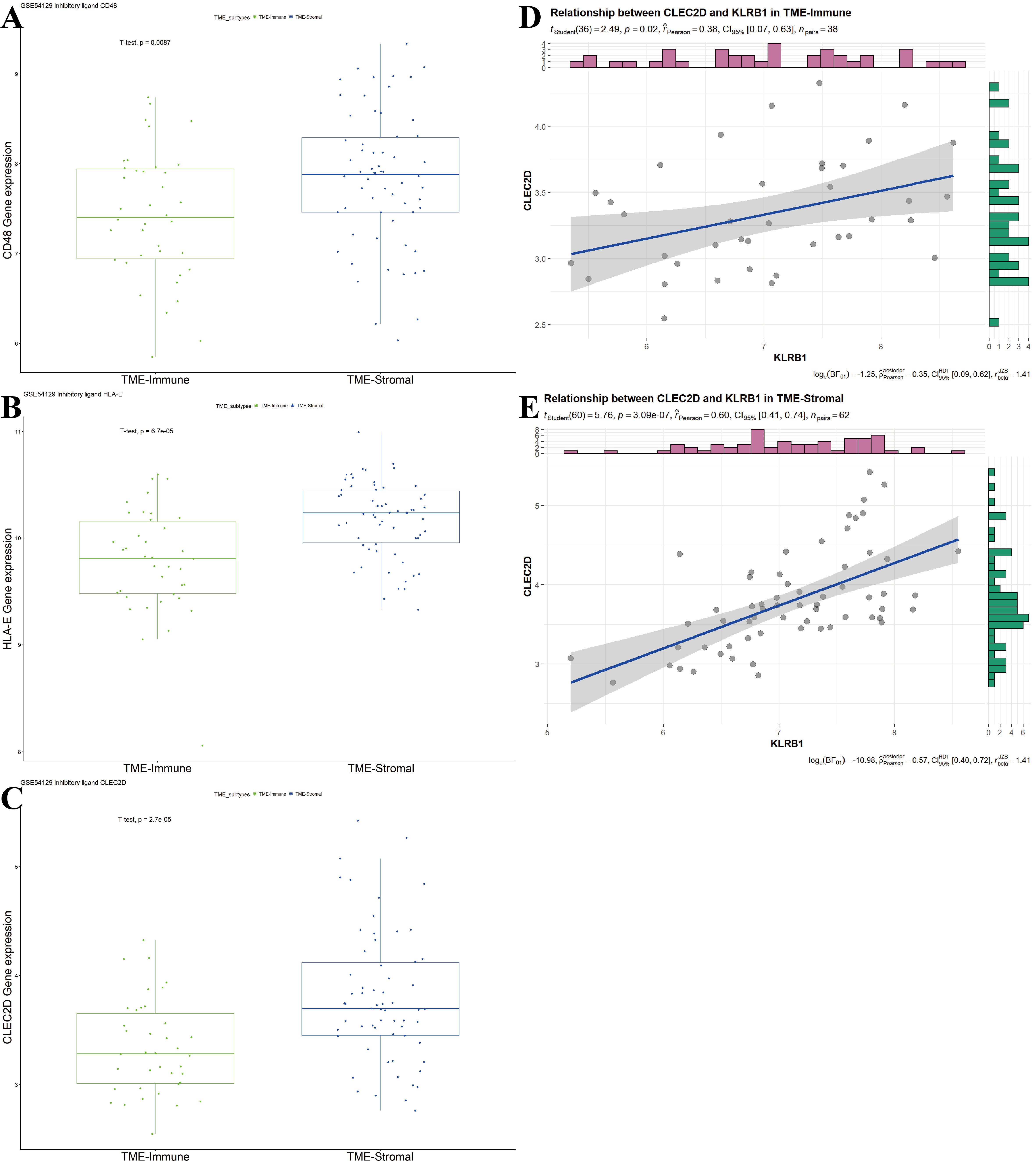

Supplement: Supplementary Figure 7 — The inhibitory receptors of NK cells (A-C) The boxplot of inhibitory receptors of NK cells. (D, E) Histogram of correlation between inhibitory receptors and ligands of NK cells in TME-Stomal and TME-Immune subtypes. [file Image_7.tif]
